# Beneficial Effects of Organosulfur Compounds from *Allium cepa* on Gut Health: A Systematic Review

**DOI:** 10.3390/foods10081680

**Published:** 2021-07-21

**Authors:** Enrique Guillamón, Pedro Andreo-Martínez, Nuria Mut-Salud, Juristo Fonollá, Alberto Baños

**Affiliations:** 1 DMC Research Center, Camino de Jayena, 82, 18620 Granada, Spain; eguillamon@domca.com (E.G.); nmsalud@dmcrc.com (N.M.-S.); juristo@ugr.es (J.F.); 2 Department of Agricultural Chemistry, Faculty of Chemistry, Campus of Espinardo, University of Murcia, 30100 Murcia, Spain; pam11@um.es; 3 Department of Chemical Engineering, Faculty of Chemistry, Campus of Espinardo, University of Murcia, 30100 Murcia, Spain; 4 Department of Nutrition and Bromatology, Campus of Cartuja, University of Granada, 18071 Granada, Spain

**Keywords:** *Allium cepa*, dysbiosis, gut microbiota (GM), intestinal health, organosulfur compounds (OSCs), thiosulphonates

## Abstract

Dietary changes affect the composition and structure of gut microbiota (GM) in animals and humans. One of the beneficial effects of consuming products derived from plants is the positive influence on immunity and gastrointestinal health. Species belonging to the genus *Allium* contain many organosulfur compounds (OSCs) that have been widely studied showing their biological properties and beneficial effects on intestinal health and GM. This is the first systematic review of OSCs from *Allium* performed following the Preferred Reporting Items for Systematic Reviews and Meta-Analyses (PRISMA) guidelines, and it is based on the evidence that we found in literature about the benefits on the GM and intestinal health demonstrated by OSCs from *Allium*, and specifically from onion. OSCs from *Allium cepa* have shown a significant antibacterial activity against a broad spectrum of antibiotic-resistant Gram-positive and Gram-negative bacteria. In addition, the intake of OSCs from onion was able to modulate the composition of GM, increasing the beneficial bacterial populations in animal models. Moreover, the beneficial effects observed in murine models of colitis suggest that these compounds could be suitable candidates for the treatment of inflammatory bowel disease (IBD) or reverse the dysbiosis caused by a high-fat diet (HFD). Despite the evidence found both *in vitro* and *in vivo*, we have not found any article that tested OSCs different from allicin in clinical trials or dietary intervention studies in humans. In this sense, it would be interesting to conduct new research that tests the benefits of these compounds in human GM.

## 1. Introduction

From the moment of their birth, humans are already colonised by microorganisms in the gastrointestinal tract (GT), oral cavity, vagina or skin. Gut microbiota (GM) includes the set of bacteria that colonise the GT and maintains the integrity of epithelium, establishing a physical barrier against pathogens. In addition, to ease digestion and absorption of nutrients, the GM helps metabolism by synthesis of essential nutrients [1] and also stimulates the production of antimicrobial compounds in the host. In recent years, plenty of assays have demonstrated that enteric bacteria play a fundamental role in aspects of human health such as the promotion of adaptive immunity, cognition pathologies, inflammatory bowel disease (IBD), metabolic syndrome or colorectal cancer [2,3,4,5,6,7,8]. Even host emotions and behaviour have been associated with changes in the ecology of their GM [9,10]. In fact, the gut microbiome is currently considered as an independent organ that shows both physiological and pathological effects [11,12].

The composition and development of the newborn’s GM are influenced by several aspects, including sex, the gestational age or drug use during the perinatal period [13]. Throughout the life of the individual, the symbiotic relationship of GM with the host can be commensal or mutualistic, including a range of factors affecting the composition of GM. There is evidence on the possible coevolution of the host and its autochthonous microbiota since this symbiotic association between host and microbiome could have played an important role in the process of human evolution [14,15]. The GM of mammals encompasses more than 100 billion microorganisms, including bacteria, eukaryotes cells such as certain fungi, archaeas and viruses. More than 90% of the bacteria in GM belong to the phylums *Firmicutes*, *Bacteroidetes*, Proteobacteria, *Actinobacteria*, *Fusobacteria* and *Verrucomicrobia*, being the two most abundant *Firmicutes* (F) and *Bacteroidetes* (B). Most of the beneficial bacteria present in GM, such as *Lactobacillus* and *Bifidobacterium*, are included in *Firmicutes* phylum [10,16]. Imbalances in the composition of GM, also known as dysbiosis, can be caused by DNA mutations, antibiotic therapy, inflammatory processes or the lifestyle, in particular, due to unhealthy eating habits and sedentary behaviours. During dysbiosis, some genera or species of potentially harmful bacteria increase in the GM, leading to a disease-prone condition, such as respiratory, cardiovascular, neurological or gastroenterological disorders [1,17,18]. For instance, obese people have a higher F/B ratio compared to people with normal weight, which implies that also obesity could also be related to changes in GM [19].

According to previous studies conducted in both humans and animals, changes in GM can occur rapidly, even within a few hours. These changes alter the functions of microorganisms and are usually characterized by a decrease in diversity and a higher F/B ratio [15], which may lead to chronic metabolic diseases. A well-studied case is the relationship between GM of newborns and gestational diabetes mellitus [20]. Other clinical and preclinical studies have shown that the presence of some bacteria belonging to the *Proteobacteria* phylum, such as *Escherichia coli*, *Helicobacter pylori* and *Salmonella enterica*, can be associated with colon inflammation and colorectal cancer [1]. On the contrary, there is evidence that the depletion of some species in GM, such as *Faecalibacterium prausnitzii*, might be related to the development of IBD [21].

Both the standard nutrient intake and severe dietary changes affect the composition and structure of GM. Diet-induced dysbiosis is a contributing factor to the development of diseases such as allergy, type 2 diabetes, cardiovascular disorders, IBD, Crohn’s disease, ulcerative colitis, irritable bowel syndrome, nonalcoholic fatty liver disease and colorectal cancer [3,22,23,24,25,26,27,28]. However, as recent studies suggest, nutritional interventions can also modify the composition of GM in a positive manner, reducing the development of pathologies associated with inflammation such as intestinal diseases, rheumatoid arthritis, asthma and acne [29,30,31]. Short-term dietary interventions in healthy humans produce rapid and statistically significant alterations in the composition of GM, although their effects are less relevant in terms of species variability [32].

**Figure 1:**
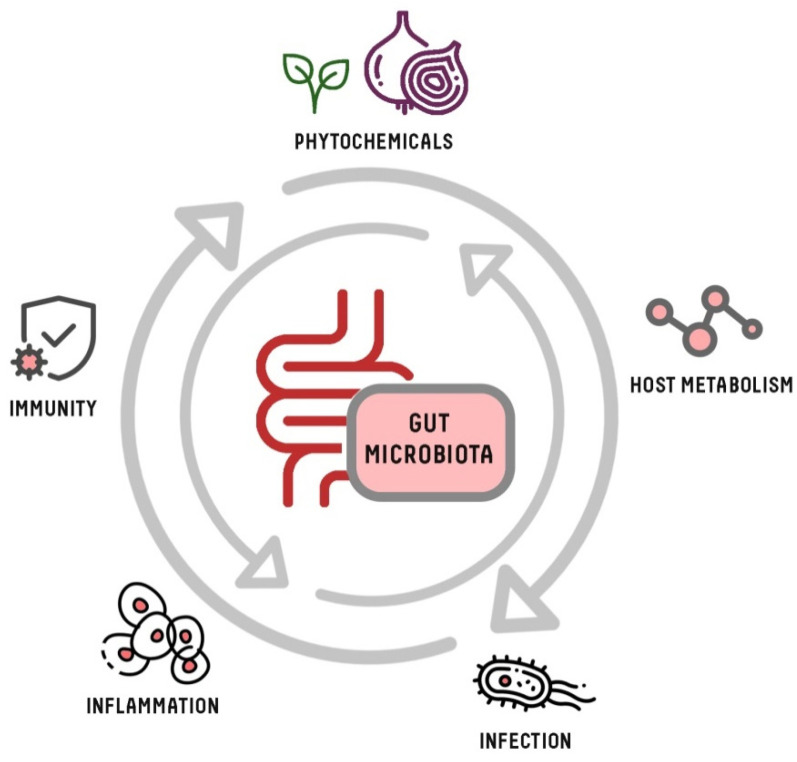
Interactions between host and gut microbiota. Phytochemicals influence the intestinal microbiota, which establishes mutual interactions and interdependencies with the host.

Dietary strategies conducted with prebiotics and probiotics are able to modulate the composition and functionality of the human GM [33]. Other dietary habits, such as high consumption of fruits and vegetables, can also significantly improve the biodiversity of GM because of their high content in bioactive phytochemicals [11,34]. Phytochemicals are natural compounds produced by plants to help them to resist fungi, bacteria and plant virus infections and also to avoid their consumption by insects or other animals [35]. The most common found in foods are polyphenols, carotenoids, phytosterols, alkaloids, glucosinolates, terpenes and organosulfur compounds (OSCs) [36]. As shown in Figure 1, phytochemicals may also affect the composition and structure of GM, and consequently impact the host metabolism, inflammatory response or development of infections.

The GM plays a key role in the general health condition, having a symbiotic relationship with the host with a direct influence on numerous diseases [29,37] (Figure 1). The immune system influences the proliferation and colonisation of certain microorganisms in intestinal microbiota through the production of immunoglobulins or cytokines [38]. In addition, GM regulates host metabolism, immunity and stimulation of the central nervous system [39,40]. The microorganisms of GM transform the components of the food ingested through fermentative processes, producing metabolites that can be beneficial or harmful [18]. These metabolites, in combination with other compounds present in food, modulate the host’s immune system. Reciprocally, some components of the diet can modulate the composition and functional capacity of GM, inducing the growth of probiotic bacteria [41]. In this way, diet can severely influence immunity and the progress of a possible host infection, altering the diversity, abundance and functions of GM [11,42] (Figure 1).

In recent literature, it can be found how prebiotics, probiotics or phytochemicals influence various pathologies, thus achieving an important therapeutic strategy for the prevention and treatment of human diseases [43,44]. In particular, dietary fibre and phytochemicals can change the composition of GM, inhibiting the growth of pathogens and increasing the population of beneficial bacteria. These bacteria produce some metabolites, such as short-chain fatty acids, which further modify the intestinal environment, improving glycaemic control, lipid profile and inflammation [15,45]. Phytochemicals have been shown to be capable of modulating GM in a few days after their inclusion in carefully controlled diets [1,35]. Although several studies have reported phytochemical-induced GM alterations, much remains to be investigated on the molecular mechanisms and interactions between the gut bacteria involved [35,46]. However, some clinically controlled dietary interventions have studied the effect of these compounds on the human GM, showing a positive influence on the health of the study subjects [17]. Although the most studied phytochemicals are polyphenols, other plant-derived compounds, such as OSCs from *Allium* vegetables, have also shown a significant potential effect to modify the composition of bacterial communities [47,48,49,50].

**Figure 2:**
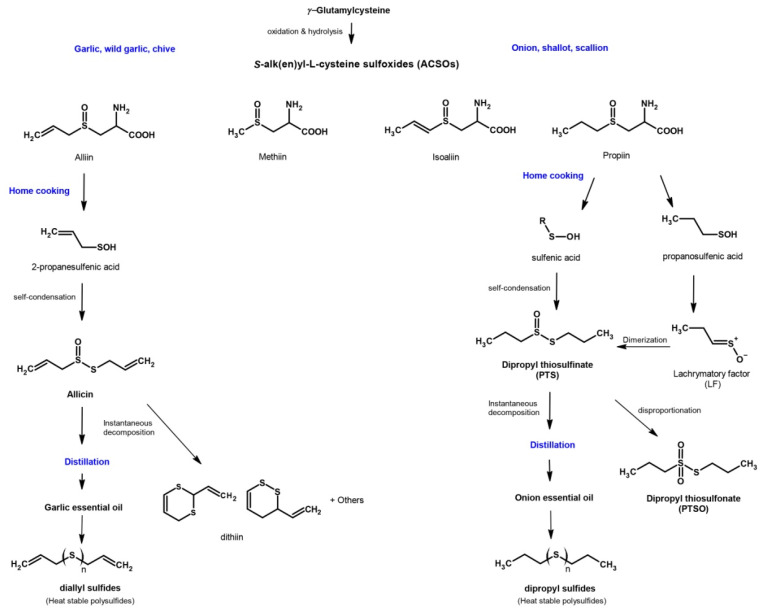
Route of formation of the main organosulfur compounds in garlic and onion.

The genus *Allium* includes more than 900 species of plants, being the most studied species *Allium sativum* (garlic), *Allium cepa* (onion), *Allium ampeloprasum var. porrum* (leek) and *Allium ascalonicum* (shallot). The therapeutic properties of these plants are widely known since time immemorial [47,48,51]. OSCs are the most important class of bioactive compounds in *Alliaceae*, which are synthesized during tissue damage in *Allium* as part of the defence mechanism against foreign aggressions. OSCs confer biological properties that are beneficial for health, such as antibacterial, antifungal, antiviral, antiprotozoal, anti-inflammatory, antidiabetic, antioxidant, antimutagenic, hepatoprotective or neuroprotective activities, among others [52,53,54,55,56,57,58]. The main OSCs in the genus *Allium* include the precursors S-alk(en)yl-L-cysteine sulphoxides (ACSO), whose transformation leads to thiosulfinates, thiosulfonates and sulfides in variable amounts and types. The characteristic pungent aroma of each *Allium* species is associated with the different levels of ACSO precursors they contain, which are primarily transformed into thiosulfinate compounds. When *Allium* bulbs are crushed, an enzyme called alliinase, common to all *Allium* species, is released from the vacuoles of cells and catalyses the cleavage of ACSO into sulfenic acid intermediates [59]. These intermediates are highly reactive and produce thiosulfinates, as shown in Figure 2.

Nevertheless, thiosulfinates are also highly reactive molecules and, under certain conditions, can decompose to form other sulfur compounds, including symmetric or asymmetric thiosulfonates and sulfides [56,57]. Depending on the *Allium* species, the pathways for the transformation of thiosulfinates and the derivatives formed may differ. In the case of garlic, alliin (S-allyl-L-cysteine sulfoxide) is the most important precursor. It is distributed into the cellular cytoplasm and, when the garlic is chopped or crushed, the contact of alliin and alliinase produces allyl sulfenic acid and dihydroalanine. Two molecules of allyl sulfenic acid are condensed, forming allicin (diallyl thiosulfinate), which is the most important OSC of the genus [56,57]. However, allicin is extremely unstable and, at room temperature, is rapidly transformed by different mechanisms in other compounds such as ajoene, dithiins or diallyl disulfide. In onion, the most common sulfur compounds are isoalliin (S-propenyl-L-cysteine sulfoxide) that changes into lachrymatory factor (Z-propanethial S-oxide); methiin (S-methyl-L-cysteine sulfoxide), also found to be present in garlic; and propiin (S-propyl-L-cysteine sulfoxide) that, due to the action of alliinase, leads to dipropyl thiosulfinate (PTS) [60]. Although PTS is more stable than allicin, it is also a labile compound that, as happens with other thiosulfinates, is transformed into dipropyl disulfide and propyl-propane thiosulfonate (PTSO) through dismutation or disproportionation reactions [56] (Figure 2).

Although numerous studies on the effects of allicin in human GM have been carried out, no articles have been found explaining their effects considering biological active OSCs derived from *Allium*, as thiosulfonates. Therefore, this is the first systematic review considering the benefits on the GM and intestinal health demonstrated by OSCs from *Allium*, and specifically from onion.

## 2. Materials and Methods

The design of the present systematic review followed the PRISMA 2020 guidelines [61]. The eligibility criteria were: Inclusion criteria: (1) works that relate the effect of OSCs from onion on GM; (2) articles published from inception to 14 March 2021; and (3) articles reporting comprehensive results and/or information on GM and OSCs. Exclusion criteria: (1) unsystematic narrative reviews; (2) works published in a language other than English; (3) dissertations and proceedings of conferences; (4) books and book chapters; (5) editorial material; (6) articles dealing with OSCs but not studying the GM; (7) articles on other diseases or plants or food or using other phytochemicals. The Boolean strings chosen were: (onion* OR thiosulfinate OR thiosulfonate OR organosulfur) AND (gut* OR intestine* OR bowel* OR gastrointestinal*) AND (microbiota* OR microflora* OR bacteria* OR microbiome* OR flora* OR bacterial* OR bacteria* OR microorganism* OR faeces* OR stool*). The four comprehensive databases used were: Scopus, Web of Science, Science Database and PubMed. The searches included works published in all languages. Scopus database options search were: “title, abstract and keywords”. Web of Science database option search was “theme” in all databases. Science Database and PubMed database option search was “all fields”.

Two authors (PA-M and EG) formed the review team in order to implement measures to minimize random errors and bias at all review stages and, separately, screened titles, abstracts, and full texts of the works for potential inclusion. The 2 reviewers evaluated them according to the eligibility criteria. Disagreements on whether a given reference should be included were resolved through discussion between the review team. Once the number of studies included for this systematic review was obtained, their quality assessment started. It consisted of an assessment of the risks of bias of each of the studies, following the model published in previous studies [10,13].

## 3. Results

### 3.1. Study Selection

The 530 works obtained by the 4 databases were crossed with the EndNote X9 software to detect possible duplicated works. A total of 140 works were eliminated at this stage. After reviewing the abstract of each of the remaining articles, those that were related to the subject of the study (238) were selected. It is worthy of mentioning that some articles can be included in more than one elimination group; however, the final criterion was agreed upon by the review team.

**Figure 3:**
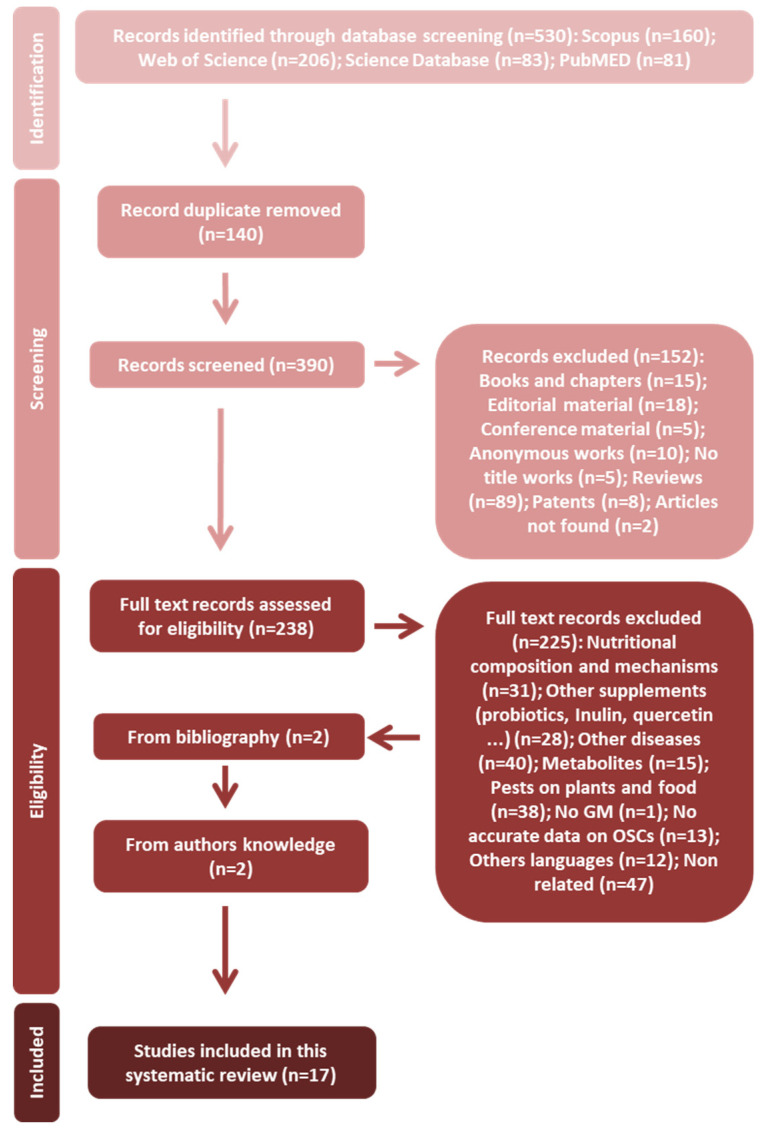
PRISMA flowchart of article selection.

Two additional articles were also found to be eligible from the bibliography section of the 238 pre-selected works. Two extra articles not identified by the electronic databases were added as the authors were aware of their existence. Finally, as shown in Figure 3, a total of 17 articles [62,63,64,65,66,67,68,69,70,71,72,73,74,75,76,77,78] were found to be eligible for the present systematic review following the full-text eligibility assessment. The characteristics of the selected articles are summarized in Table 1. This table collects an overview of these articles, which includes the type of *Allium* product used (OSCs or raw extract), the doses applied, evaluation model (*in vitro* or *in vivo*), the length of the study and the main findings related to their beneficial effects in gut health (Table 1).

### 3.2. Evidence from In Vitro Assays

In this review, it was found that two articles tested *Allium* products in *Clostridium difficile* populations [63,64]. The results showed how these products exerted a bactericidal effect on different types of pathogenic bacteria. One article reported the *in vitro* efficacy of one thiosulfonate in two human tumour cells [67]. This compound showed its role in the anti-inflammatory response of both cell lines tested and the analysis of the results was made through the analysis of relevant mediators in the pathogenesis of IBD. Another article evaluated the *in vitro* influence of OSCs in GM of pigs, reporting the reduction of harmful bacteria [65].

### 3.3. Evidence from In Vivo Assays

Five of the selected articles reported results of assays conducted with Allium products in murine models. Vezza et al. [67] investigated the effects of one onion-derived OSC in two models with mice with colitis, which were able to modulate the GM composition of the treated mice. Two articles studied how the consumption of an OSC derived from garlic (alliin) influenced the GM in healthy rats [68], as well as in mice with obesity induced by a high-fat diet (HFD) [66]. The results showed that, in both cases, the gut health of the treated animals improved following changes in their GM populations. The other two articles conducted in mice studied the effect of garlic extracts in mice with HFD-induced dyslipidemia. Both articles reported optimal results in treated animals, achieving an increase in beneficial bacteria of the GM [62,69]. Six articles of this systematic review dealt with trials conducted in birds. A total of four of them were carried out with broiler chickens [70,71,72,73] and two with laying hens [74,76]. In all cases, it was observed an increase of certain beneficial bacteria and a decrease of harmful bacteria populations in the guts of the evaluated animals. Three articles reported the influence of Allium products intake in swine, showing beneficial effects such as the increase in beneficial bacteria [75,77] or the reduction of pathogenic species [78] in the GM. All the assays conducted in birds and swine models showed how the animals fed with diets supplemented with Allium products had an improvement in their intestinal health, including an important antibacterial activity against pathogenic species. Finally, no article was found dealing with clinical trials or nutritional intervention studies in humans.

### 3.4. Risk of Bias

As discussed in Section 2, the risks of bias of each study were measured following the model published in former studies [10]. Studies were considered to have a high (1–6), medium (7–9) or low (10–12) risk of bias in terms of the consolidated score out of 12. A low risk of bias was found in 14 articles selected in the present systematic review, and a moderate risk was found in 3 articles. None of the selected articles showed a high risk of bias. When assessing the quality of selection, the studies present more limitations in comparability and in other bias items (controlled dietary intake, comorbidity, etc.). Full details of scoring across the three types of bias are given in Table 2.

### 3.5. Limitations

The present systematic review was limited, by definition, by the databases used, the Boolean strings chosen and the established criteria for inclusion or exclusion. In other words, limitations related to search strategy and methodology. For instance, three of the articles discussed [65,66,78] were not found by the four electronic databases used as they did not contain the keywords of the Boolean string chosen in their title, abstract, or keywords sections. However, the search strategy was deeply exhaustive, thus it was expected that only relatively few relevant studies were not identified. Only works reported in English were included and this fact could suggest bias in the search, as reported elsewhere [79,80]. In addition, the fact that 17 articles were finally included could suggest bias in the eligibility strategy as the final sample of selected articles could be not representative [79]. Finally, the lack of sufficient statistical information reported in the selected articles made it impossible to combine the results of different studies in a meta-analysis.

## 4. Discussion

The present systematic review aims to discuss 17 articles that have studied the relationship between the consumption of *Allium* products, intestinal health and GM. This work is focused on the influence of the OSCs present in *Allium* species, although there are also some studies that describe beneficial effects on GM of non-sulfur compounds present in the genus *Allium*. For example, the oral administration of quercetin together with resveratrol was able to restore the dysbiosis of the GM induced by HFD in Wistar rats [81]. The results demonstrated that the treatment could modulate the GM of rats, decreasing the population of *Firmicutes* and inhibiting the relative abundance of obesity-related families such as *Coriobacteriaceae*, *Lachnospiraceae*, *Acidaminococcaceae*, *Desulfovibrionaceae* and *Bilophila*. In another recent study, dietary supplementation with a polysaccharide from Jinxiang garlic alleviated colitis in mice. The treated animals showed an improvement in the structure of the intestinal mucosa, and, in addition, the blocking of certain pro-inflammatory cytokines was observed [82]. We have not found any other systematic review linking OSCs to gut health or GM, except a systematic review and meta-analysis that evaluated solely the use of allicin as a complementary therapy for *H. pylori* infection and whose efficacy was evaluated in randomized controlled trials [83].

Beyond allicin, other OSCs from *Allium* have shown antibacterial activity against Gram-positive and Gram-negative bacteria, including species of the genera *Escherichia*, *Salmonella*, *Bacteroidetes*, *Klebsiella*, *Streptococcus*, *Neisseria*, *Proteus*, *Clostridium*, *Mycobacterium*, *Staphylococcus*, *Micrococcus* and *Bacillus* [52]. Specifically, thiosulfinates and thiosulfonates have significant antimicrobial activity against *Escherichia*, *Salmonella*, *Clostridium*, *Campylobacter* and *Aspergillus* species [70], and also even against numerous strains of antibiotic-resistant bacteria isolated from human clinical samples [52].

OSCs from *Allium* have also shown antibacterial effects against other bacteria associated with severe gastrointestinal symptoms such as *C. difficile*. This bacterium can cause symptoms ranging from diarrhoea to life-threatening inflammation of the colon. Toxins are the main virulence factor that initiates *C. difficile* infection (CDI), causing inflammation and damaging the lining of the intestine. CDI most often affects older adults with long-term healthcare treatment and usually occurs after using antibiotics. For this reason, it could be important to find alternatives to antibiotics for the prevention and treatment of this disorder [63,64]. In a first study conducted by Roshan et al. [63], several natural products were tested against *C. difficile*, with the highest activity exerted by the juice extracted from fresh garlic cloves. The great advantage of using OSCs from *Allium* against CDI is that these compounds have a bactericidal effect against *C. difficile* at lower concentrations than those that affect beneficial bacteria such as *Lactobacillus* species. In a later study conducted by the same researcher team [64], fresh onion extract showed a reduction not only in *C. difficile* but also in the production of its pathogenic toxins, both in Vero (monkey kidney cells) and in HT-29 (human colorectal adenocarcinoma cells) cell lines.

Another species of the same genus, *Clostridium perfringens*, is part of the normal GM of livestock animals, mainly in poultry species such as broilers or turkeys. This bacterium grows under anaerobic conditions and produces toxic spores highly resistant to drying, heat, acids and other harsh environmental factors. Under normal health conditions, beneficial bacteria in the gut keep *C. perfringens* counts small in numbers, thus that it does not cause any disease in animals. However, when conditions change in the GT, the population of this bacteria increases, and the production of toxins may lead to the appearance of intestinal irritation and a disease known as necrotic enteritis, which severely affects the poultry industry [84]. The banning of the use of antibiotic growth promoters (AGP) in animal feed resurfaced this issue, increasing the number of flocks affected and, consequently, the economic losses [85]. Therefore, the inclusion of antimicrobial phytochemicals such as OSCs into feed has become a useful approach to reduce pathogenic bacteria populations in the GM and the reduction of losses caused by the aforementioned disease [86].

The antimicrobial activity of *Allium* extracts containing PTS and PTSO has also been evaluated in an *in vitro* assay conducted by Ruiz et al. with GM of sows [65]. The results showed significant antibacterial activity of both compounds against all the studied groups, being *Enterobacteriaceae* the most sensitive species. In addition, the same authors carried out the kill curves of PTS and PTSO against *E. coli* and *Salmonella typhimurium*, two common enteropathogens in swine production. Both OSCs exerted a rapid bactericidal effect in a dose-dependent manner. On the contrary, *Lactobacillus* and *Bifidobacterium* were the most resistant populations to these compounds. In line with these results, other articles reported the antibacterial activity of PTS and, especially, PTSO, against Gram-positive and Gram-negative human clinical isolates [52,87]. The results highlighted the special sensitivity of *E. coli* or *Salmonella* strains, regardless of their resistance to antibiotics such as cefotaxime or cefazidime, or their capacity to produce Extended-Spectrum Beta-Lactamase. Moreover, the activity against Gram-positive bacteria such as methicillin-resistant *Staphylococcus aureus*, *Enterococcus faecalis* and *Streptococcus agalactiae* was higher than the one observed against enterobacteria, especially in the case of PTSO [52,87].

There is much evidence that the condition of the gut microbiome is deeply involved in animal and human health status. Bacterial species belonging to the *Lactobacillus* and *Bifidobacterium* genera are generally considered beneficial, while the increase of *Clostridum*, *Eubacterium* or *Bacteroidetes*, is considered harmful to health [88,89]. Variations in the composition and function of the GM lead to a decrease in microbial diversity and the expansion of certain bacterial groups usually considered as harmful, which is related to the development of many diseases [88,89,90,91].

In two studies carry out in mice with dyslipidemia induced by HFD, the administration of *Allium* products derived from garlic showed an attenuation of the harmful effects caused by this diet on GM [62,69]. In one of these assays, whole garlic was administered [69], while, in the other one, the *Allium* product tested was an allicin-free garlic extract (AFG) [62]. In both assays, diets supplemented with garlic positively impacted GM, as supplementation succeeded in restoring the number of beneficial bacteria to the gut health of mice, such as *Lachnospiraceae* and *Akkermansia*, whose counts had been reduced by HFD. The administration of AFG extract was able to reduce the increase in the F/B ratio caused by HFD, relieving the negative impact of this diet on the health of animals and their inflammatory response [62]. The mice that consumed whole garlic in the latter study, showed a greater regulation of the diversity of GM, which led to revert the reduction of *Bacteroidetes* and beneficial species of the genus *Prevotella* caused by HFD [69].

In another study conducted in mice with obesity induced by HFD, the effect of consuming alliin was evaluated in order to demonstrate the preventive effect of this compound against the metabolic risk factors associated with obesity [66]. The results showed an improvement in glucose homeostasis and lipid profile in the animals whose diet was supplemented with alliin. The mechanism for the hypoglycemic effect of this compound is not clear, though it might be partially attributed to the modulating effect of alliin on the composition of GM, and, in particular, to the increase of *Ruminococcaceae*, which seems to inhibit the negative effects caused by HFDs. Beyond *Ruminococcaceae*, mice fed with supplemented diet experienced an increase in other classes of *Firmicutes* and also *Actinobacteria*, along with a reduction in the number of *Bacteroidetes* and *Proteobacteria*, though these results did not reach a significant level [66]. Similar findings were obtained in another study conducted with alliin in healthy rats [68]. The group that took alliin presented a higher abundance of *Firmicutes* and a lower abundance of *Bacteroidetes* than the control group, although the differences were not statistically significant [68].

Vezza et al. [67] tested the activity of PTSO in two experimental models of mice with colitis. Microbial richness and evenness were significantly decreased in the colitic group compared to the non-colitic one, being both ecological parameters restored in colitic mice treated with PTSO. In particular, the composition of GM at the phylum level showed a significantly higher abundance of *Firmicutes* and a lower level of *Bacteriodetes* in the colitic control group when compared with non-colitic mice. Oral administration of PTSO to colitic mice was able to ameliorate these changes, having a decrease of *Firmicutes* abundance in the gut of the mice that led to a reduction in the F/B ratio. On the contrary, although the level of *Bacteriodetes* was not significantly modified, the abundance of *Actinobacteria* increased in the PTSO-treated colitic mice compared to the control group. The authors concluded that the administration of PTSO to colitic mice was able to restore the parameters in the colitic models without showing statistical differences with non-colitic mice [67].

The rising global restrictions in the use of AGP worldwide have increased the need to apply new antibacterial or prebiotic ingredients for feed. Among these ingredients, phytochemicals stand out for their antimicrobial activity and their ability to modulate GM [92,93,94]. In particular, there are several references about the benefits of the inclusion of secondary metabolites from *Allium* species in the diet of animals. Among these benefits, it can be highlighted the improvement of intestinal health, the modulation of the inflammatory response and the improvement in the absorption of nutrients, which, consequently, speeds the growth of animals [92,95,96,97].

There is much evidence that the state of GM in poultry and swine contributes to their health and productivity [98,99]. The influence of the microbiome is particularly relevant in young animals since the composition of their GM is still under development [16]. According to Rubio et al. [72], *Bacteroidetes* family is involved in important metabolic activities in broilers, including the metabolism of carbohydrates and nitrogenous substances, as well as the prevention of the colonization of pathogens such as *Salmonella*, *Campylobacter jejuni* and *C. perfringens*. Other families involved in the composition of the cecal microbiota of broilers are *Lachnospiraceae*, *Ruminococcaceae* and *Micrococcaceae* [72]. Nevertheless, GM of swine is dominated by *Firmicutes*, followed by *Proteobacteria* in the large intestine and *Bacteroidetes* in the small intestine [100].

It has been reported that supplementation with *Allium hookeri* leaves produced modulation of GM in broilers [101]. In addition, the addition of onion powder to the diet of broilers caused a significant reduction in *E. coli* population and the increase of *Lactobacillus* and *Streptococcus* species, achieving an improvement in the gut histomorphology [96]. However, most of the research on the effects of OSCs on GM of birds has been conducted with the use of specific secondary metabolites from onion, as PTSO. For example, Ruiz et al. [65] reported that PTSO is able to modulate the GM composition associated with the ileal mucosa of *Bifidobacterium* species and the *Blautia coccoides*/*Eubacterium rectole* bacterial group of broiler chickens. Other positive data from this study were the increase of certain species of *Bifidobacterium* in the gut, such as *B. pseudolongum*, *B. longum* and *B. pseudocatenulatum*. In another recent article [74], the use of an onion extract containing PTSO was also able to modify GM of laying hens, reducing the number of *Enterobacteriaceae*, *Lactobacillus* and *Bifidobaterium* in faeces after one week of supplementation. In two studies conducted with broilers [70,71], the administration of an *Allium* product containing PTS and PTSO improved the gut health of the treated animals. In one of these assays, PTS and PTSO showed antimicrobial activity against different bacterial groups, reducing the fecal counts of *Enterobacteriaceae* and coliforms [70]. In the second study referred, PTSO supplementation modulated the GM composition in broiler chickens and also improved the nutrient digestibility without affecting activity of mucosal enzymes [71]. Similar results were obtained in another assay with broilers [82], where a significant bactericidal effect of PTSO against enterobacteria, coliforms, *E. coli*, *C. jejuni* and *Salmonella*, both *in vitro* and *in vivo*, was revealed.

Results showing the benefits of OSCs have also been reported in experiments in swine. Sanchez et al. [75] have shown that the inclusion of PTSO in the growing-finishing pig diet was beneficial for their GM. The authors found a significant increase of *Lactobacillus* and a reduction in the counts of coliforms and enteropathogens (*Salmonella* and *Clostridium*) in faeces, compared to those animals that consumed a basal diet that included antibiotics but not PTSO. The analysis of microbiome in sows, reported in another recent article [78], showed the reduction of intestinal pathogenic species in those animals that ingested an extract composed of garlic and probiotics, compared to the control group. Moreover, in another assay conducted in swine [65], another extract containing PTS and PTSO was used as a feed additive. The results revealed an improvement in the intestinal health of the treated animals, increasing the digestibility of the nutrients.

Taking into account the results mentioned above, it could be concluded that PTSO, when added to the diet of the animals, is capable of reducing certain GM bacterial populations in a dose-dependent manner, being enterobacteria and coliforms the most affected. In another and more recent article, the effect of the intake of an onion and garlic extract including PTS and PTSO was evaluated on the GM of piglets [77]. In this trial, the bacteria diversity in different intestinal regions was analyzed. These data were compared to the gut composition of a group of piglets whose diet was supplemented with antibiotics (colistin and zinc oxide) and another control group. The microbiome of piglets belonging to the *Allium* group and the control group were very similar. On the contrary, the GM of piglets belonging to the antibiotic group showed a lower proportion of bacilli and a higher proportion of Clostridia and Bacteroidia. More specifically, the number of *Lactobacillus* decreased in these animals, especially in the colon, while the genus *Prevotella* increased [77].

Further experiments are necessary to deepen our understanding of the interactions between GM and the host, as well as the mechanisms of action involved. Future studies should try to homogenize, as far as possible, the characteristics of samples to be compared and the methods for detecting bacterial populations. However, given the numerous pieces of evidences discussed, both *in vitro* and in experimental models, the benefits for GM of treatments with OSCs seem clear. However, it would be interesting to perform dietary interventional assays with these products in humans since none has been made until the date with compounds derived from onion such as PTS or PTSO.

## 5. Conclusions

This is the first systematic review about the benefits on the GM and intestinal health by *Allium* products, specifically by secondary metabolites from onion. A total of 530 publications were found from the four different electronic databases used. This number was reduced to thirteen after removing duplicate publications and the application of the inclusion/exclusion criteria established by the reviewer team. In addition, two articles were also found to be eligible from the bibliography section of the pre-selected works, and two extra articles, not identified by the electronic databases, were added as the authors were aware of their existence. Taking into account all of the aforementioned, 17 original articles were included in this systematic review to describe the current scientific evidence of the modulating effect on GM exerted by *Allium* products and, in particular, by OSCs from *Allium cepa*. These compounds showed significant antibacterial activity against a broad spectrum of enteric pathogens, both *in vitro* and *in vivo*, including antibiotic-resistant bacteria isolated from clinical specimens. Numerous *in vivo* assays, conducted with different animal models, reported that the intake of OSCs from onion was able to modulate the composition of GM, increasing the beneficial bacterial populations. Moreover, the effects of these compounds in murine models with colitis and obesity suggested that they could be suitable candidates for the treatment of IBD or reverse the alteration of GM caused by HFDs. Despite the evidence found in different animal models, no article has been found reporting the effects of other OSCs, apart from allicin, in clinical trials or dietary interventions in humans. In this sense, it would be interesting to conduct new research to test the benefits of these compounds in human GM.

## Figures and Tables

**Table 1:** Overview of studies reporting the evaluation of OSCs from *Allium* on gut health.

Study (Author, Year)	*Allium* Product	Dose	Model	Time	Main Findings
Roshan et al., 2017 [63]	Onion juice	Raw material	*C. difficile*	24–48 h	↓ *C. difficile*
Roshan et al., 2018 [64]	Fresh onion bulb extract	Raw material	Toxin production by *C. difficile* in Vero and HT-29 cells	48 h	↓ Toxin production and activity at 12.5%
Ruiz et al., 2010 [65]	PTS ^1^ and PTSO ^2^	50, 200 and 400 mg/kg	Gut microbiota of swine	24, 48, 72 h	↓ *E. coli* and *S. typhimurium* = *Lactobacillus* and *Bifidobacterium*
Zhai, et al., 2018 [66]	Alliin	0.1 mg/mL	C57BL/6J DIO ^3^ mice	8 weeks	↑ *Actinobacteria* and *Firmicutes* ↓ *Bacteroidetes* and Proteobacteria
Vezza et al., 2018 [67]	PTSO	0.02–4.5 mg/L 0.01, 0.1, 0.5, 1, 10 mg/kg	Caco-2, THP-1 cellsMice with colitis	24 h 10 days	↓ Pro-inflammatory cytokines ↓ Firmicutes in the gut contents ↓ Actinobacteria
Zhang et al., 2019 [68]	Alliin	80 mg/kg	Rats	14 days	↑ *Firmicutes* ↑ *Allobaculum* ↓ *Bacteroidetes* and *Candidatus*
Chen et al., 2019 [69]	Garlic extract	Raw material	C57BL/6N mice	12 weeks	↑ α-diversity ↑ Lachnospiraceae ↓ *Prevotella*
Chen et al., 2020 [62]	AFG ^4^ extract	10.000–50.000 mg/kg	C57BL/6N mice	11 weeks	↓ F/B ^5^ en HFD ^6^ ↓ Dorea ↑ Lachnospiraceae ↑ *Lactobacillus*
Peinado et al., 2012 [70]	PTSO	45–135 mg/kg	broiler chickens	13 days	↓ *Salmonella* ↓ *Campylobacter*
Peinado et al., 2013 [71]	PTSO	45–90 mg/kg	Broiler chickens	21 days	↓ *C. coccoides* ↓ *C. leptum* ↑ *Bacteroidetes* in the ileal contents ↓ *Bacteroidetes* in the cecal contents
Rubio et al., 2015 [72]	PTSO	45–90 mg/kg	Broiler chickens	21 days	↑ *Bacteroidetes* ↓ *Escherichia*–*Shigella*
Ruiz et al., 2015 [73]	PTSO	90 mg/kg	Broiler chickens	21 days	↑ *Bifidobacterium* in ileal mucosal
Abad et al., 2020 [74]	PTSO	30 mg/kg	Laying hens	28 days	↑ *Lactobacillus* and *Bifidobacterium* ↓ Enterobacteriaceae
Sánchez et al.,2020 [75]	*Allium* extract	5 g/kg	Growing-finishing pigs	103 days	↓ *Salmonella* ↑ *Lactobacillus* in faeces ↑ Levels of propionic, isobutyric and isovaleric acids in faeces
Satora et al., 2020 [78]	Garlic extract	10 mL	Sows	From 80th day of gestation to weaning day	↓ Pathogenic bacteria
Rabelo et al., 2021a [76]	PTSO	60 mg/kg	Laying hens	30 days	↑ *Lactococcus* in the ileum ↑ *Lactobacillus* in the cecum
Rabelo et al., 2021b [77]	*Allium* extract	20 mg/kg	Piglets	42 days	↓ α-diversity in caecum and colon ↑ Bacilli and ↓ Bacteroidia in caecum

^1^ Propyl propane thiosulfinate (PTS); ^2^ Propyl propane thiosulfonate (PTSO); ^3^ diet induced obese (DIO); ^4^ Allicin-free garlic (AFG) extract; ^5^ *Firmicutes*/*Bacteroidetes* (F/B); ^6^ high-fat diet (HFD).

**Table 2:** Risk of bias for the methodological quality of studies on organosulfur compounds and gut microbiota.

Study	Clear Objetive	Adequate Sample Size	Identification and Evaluation of Sample	Comparability	Other Biases (Controlled Dietary Intake, Comorbidity…)	Adequate Statistical Analysis	Total	Risk of Bias	General Risk of Bias
Chen et al., 2019 [69]	2	2	2	1	2	1	10	2	L
Peinado et al., 2012 [70]	2	2	2	1	1	2	10	2	L
Peinado et al., 2013 [71]	2	2	2	1	1	2	10	2	L
Rubio et al., 2015 [72]	2	2	2	1	1	2	10	2	L
Ruiz et al., 2015 [73]	2	2	2	1	1	2	10	2	L
Roshan et al., 2017 [63]	2	1	2	1	1	2	9	3	M
Roshan et al., 2018 [64]	2	1	2	1	1	2	9	3	M
Vezza et al., 2019 [67]	2	2	2	2	1	2	11	1	L
Zhang et al., 2019 [68]	2	2	2	1	2	2	11	1	L
Chen et al., 2020 [62]	2	2	2	1	1	2	10	2	L
Sánchez et al., 2020 [75]	2	2	2	1	2	2	11	1	L
Zhai et al., 2018 [66]	2	2	2	1	2	2	11	1	L
Ruiz et al., 2010 [65]	2	1	1	1	1	2	8	4	M
Rabelo et al., 2021a [76]	2	2	2	2	1	2	11	1	L
Rabelo et al., 2021b [77]	2	2	2	2	1	2	11	1	L
Abad et al., 2021 [74]	2	2	2	2	1	2	11	1	L
Satora et al., 2020 [78]	2	2	2	2	1	2	11	1	L

Note: 0: not reported; 1: not appropriately or clearly evaluated; 2: appropriately evaluated. M: medium (7–9); L: low (10–12); H: high (6−1).

## References

[1] Yin R., Kuo H.-C., Hudlikar R., Sargsyan D., Li S., Wang L., Wu R., Kong A.-N. (2019). Gut Microbiota, Dietary Phytochemicals, and Benefits to Human Health. Curr. Pharmacol. Rep..

[2] Wang L., Zhu L., Qin S. (2019). Gut Microbiota Modulation on Intestinal Mucosal Adaptive Immunity. J. Immunol. Res..

[3] Proctor C., Thiennimitr P., Chattipakorn N., Chattipakorn S.C. (2016). Diet, gut microbiota and cognition. Metab. Brain Dis..

[4] Jiang C., Li G., Huang P., Liu Z., Zhao B. (2017). The Gut Microbiota and Alzheimer’s Disease. J. Alzheimer’s Dis..

[5] Kataoka K. (2016). The intestinal microbiota and its role in human health and disease. J. Med. Investig..

[6] Patterson E., Ryan P.M., Cryan J.F., Dinan T.G., Ross R.P., Fitzgerald G.F., Stanton C. (2016). Gut microbiota, obesity and diabetes. Postgrad. Med. J..

[7] Ni J., Wu G.D., Albenberg L., Tomov V.T. (2017). Gut microbiota and IBD: Causation or correlation?. Nat. Rev. Gastroenterol. Hepatol..

[8] Tilg H., Adolph T.E., Gerner R.R., Moschen A.R. (2018). The Intestinal Microbiota in Colorectal Cancer. Cancer Cell.

[9] Andreo-Martínez P., Rubio-Aparicio M., Sánchez-Meca J., Veas A., Martínez-González A.E. (2021). A Meta-analysis of Gut Microbiota in Children with Autism. J. Autism Dev. Disord..

[10] Martínez P.A., García-Martínez N., Sánchez-Samper E.P., Martínez-González A.E. (2019). An approach to gut microbiota profile in children with autism spectrum disorder. Environ. Microbiol. Rep..

[11] Lai H.-C., Young J.D., Lin C.-S., Chang C.-J., Lu C.-C., Martel J., Ojcius D., Ko Y.-F. (2014). Impact of the gut microbiota, prebiotics, and probiotics on human health and disease. Biomed. J..

[12] Li X., Liu L., Cao Z., Li W., Li H., Lu C., Yang X., Liu Y. (2020). Gut microbiota as an “invisible organ” that modulates the function of drugs. Biomed. Pharmacother..

[13] Martínez-González A.E., Andreo-Martínez P. (2019). The Role of Gut Microbiota in Gastrointestinal Symptoms of Children with ASD. Medicina.

[14] Dominguez-Bello M.G., Godoy-Vitorino F., Knight R., Blaser M.J. (2019). Role of the microbiome in human development. Gut.

[15] Dey P. (2019). Gut microbiota in phytopharmacology: A comprehensive overview of concepts, reciprocal interactions, biotransformations and mode of actions. Pharmacol. Res..

[16] Maynard C., Weinkove D. (2018). The Gut Microbiota and Ageing. Prokaryotic Cytoskelet..

[17] Lynch S.V., Pedersen O. (2016). The Human Intestinal Microbiome in Health and Disease. N. Engl. J. Med..

[18] Tomasello G., Mazzola M., Leone A., Sinagra E., Zummo G., Farina F., Damiani P., Cappello F., Geagea A.G., Jurjus A. (2016). Nutrition, oxidative stress and intestinal dysbiosis: Influence of diet on gut microbiota in inflammatory bowel diseases. Biomed. Pap..

[19] Kałużna-Czaplińska J., Gątarek P., Chartrand M.S., Dadar M., Bjørklund G. (2017). Is there a relationship between intestinal microbiota, dietary compounds, and obesity?. Trends Food Sci. Technol..

[20] Su M., Nie Y., Shao R., Duan S., Jiang Y., Wang M., Xing Z., Sun Q., Liu X., Xu W. (2018). Diversified gut microbiota in newborns of mothers with gestational diabetes mellitus. PLoS ONE.

[21] Cheng D., Song J., Xie M., Song D. (2019). The bidirectional relationship between host physiology and microbiota and health benefits of probiotics: A review. Trends Food Sci. Technol..

[22] Kang Y., Cai Y., Zhang H. (2017). Gut microbiota and allergy/asthma: From pathogenesis to new therapeutic strategies. Allergol. Immunopathol..

[23] Rachid R., Chatila T. (2016). The role of the gut microbiota in food allergy. Curr. Opin. Pediatr..

[24] Sekirov I., Russell S.L., Antunes L.C.M., Finlay B.B. (2010). Gut Microbiota in Health and Disease. Physiol. Rev..

[25] Leung C., Rivera L., Furness J.B., Angus C.L.P.W. (2016). The role of the gut microbiota in NAFLD. Nat. Rev. Gastroenterol. Hepatol..

[26] Nishida A., Inoue R., Inatomi O., Bamba S., Naito Y., Andoh A. (2018). Gut microbiota in the pathogenesis of inflammatory bowel disease. Clin. J. Gastroenterol..

[27] Zhu W., Winter M.G., Byndloss M.X., Spiga L., Duerkop B.A., Hughes E.R., Büttner L., Romão E.D.L., Behrendt C.L., Lopez C.A. (2018). Precision editing of the gut microbiota ameliorates colitis. Nature.

[28] Lopez-Santamarina A., Gonzalez E., Lamas A., Mondragon A., Regal P., Miranda J. (2021). Probiotics as a Possible Strategy for the Prevention and Treatment of Allergies. A Narrative Review. Foods.

[29] Boulangé C.L., Neves A.L., Chilloux J., Nicholson J.K., Dumas M.-E. (2016). Impact of the gut microbiota on inflammation, obesity, and metabolic disease. Genome Med..

[30] Gioia C., Lucchino B., Tarsitano M.G., Iannuccelli C., Di Franco M. (2020). Dietary Habits and Nutrition in Rheumatoid Arthritis: Can Diet Influence Disease Development and Clinical Manifestations?. Nutrients.

[31] Barcik W., Boutin R.C., Sokolowska M., Finlay B.B. (2020). The Role of Lung and Gut Microbiota in the Pathology of Asthma. Immunity.

[32] David L.A., Maurice C.F., Carmody R.N., Gootenberg D.B., Button J.E., Wolfe B.E., Ling A.V., Devlin A.S., Varma Y., Fischbach M.A. (2014). Diet rapidly and reproducibly alters the human gut microbiome. Nature.

[33] Marchesi J.R., Adams D.H., Fava F., Hermes G.D.A., Hirschfield G.M., Hold G., Quraishi M.N., Kinross J., Smidt H., Tuohy K.M. (2016). The gut microbiota and host health: A new clinical frontier. Gut.

[34] Albenberg L.G., Wu G.D. (2014). Diet and the Intestinal Microbiome: Associations, Functions, and Implications for Health and Disease. Gastroenterology.

[35] Dingeo G., Brito A., Samouda H., Iddir M., La Frano M.R., Bohn T. (2020). Phytochemicals as modifiers of gut microbial communities. Food Funct..

[36] Giampieri F., Battino M. (2020). Bioactive Phytochemicals and Functional Food Ingredients in Fruits and Vegetables. Int. J. Mol. Sci..

[37] Belkaid Y., Hand T.W. (2014). Role of the Microbiota in Immunity and Inflammation. Cell.

[38] D’Amelio P., Sassi F. (2018). Gut Microbiota, Immune System, and Bone. Calcif. Tissue Int..

[39] Fung T.C., Olson C.A., Hsiao E.Y. (2017). Interactions between the microbiota, immune and nervous systems in health and disease. Nat. Neurosci..

[40] Dantzer R. (2018). Neuroimmune Interactions: From the Brain to the Immune System and Vice Versa. Physiol. Rev..

[41] Singh R.K., Chang H.-W., Yan D., Lee K.M., Ucmak D., Wong K., Abrouk M., Farahnik B., Nakamura M., Zhu T.H. (2017). Influence of diet on the gut microbiome and implications for human health. J. Transl. Med..

[42] Mentella M.C., Scaldaferri F., Pizzoferrato M., Gasbarrini A., Miggiano G.A.D., Chiara M.M., Franco S., Marco P., Antonio G., Donato M.G.A. (2020). Nutrition, IBD and Gut Microbiota: A Review. Nutrients.

[43] Sanders M.E., Merenstein D.J., Reid G., Gibson G.R., Rastall R.A. (2019). Probiotics and prebiotics in intestinal health and disease: From biology to the clinic. Nat. Rev. Gastroenterol. Hepatol..

[44] Moorthy M., Chaiyakunapruk N., Jacob S.A., Palanisamy U.D. (2020). Prebiotic potential of polyphenols, its effect on gut microbiota and anthropometric/clinical markers: A systematic review of randomised controlled trials. Trends Food Sci. Technol..

[45] Makki K., Deehan E.C., Walter J., Bäckhed F. (2018). The Impact of Dietary Fiber on Gut Microbiota in Host Health and Disease. Cell Host Microbe.

[46] Carrera-Quintanar L., Roa R.I.L., Quintero-Fabián S., Sánchez-Sánchez M.A., Vizmanos B., Ortuño-Sahagún D. (2018). Phytochemicals That Influence Gut Microbiota as Prophylactics and for the Treatment of Obesity and Inflammatory Diseases. Mediat. Inflamm..

[47] Shang A., Cao S.-Y., Xu X.-Y., Gan R.-Y., Tang G.-Y., Corke H., Mavumengwana V., Li H.-B. (2019). Bioactive Compounds and Biological Functions of Garlic (*Allium sativum* L.). Foods.

[48] Asemani Y., Zamani N., Bayat M., Amirghofran Z. (2019). Allium vegetables for possible future of cancer treatment. Phytother. Res..

[49] Petropoulos S., Di Gioia F., Ntatsi G. (2017). Vegetable Organosulfur Compounds and their Health Promoting Effects. Curr. Pharm. Des..

[50] Singh A.K., Cabral C., Kumar R., Ganguly R., Rana H.K., Gupta A., Lauro M.R., Carbone C., Reis F., Pandey A.K. (2019). Beneficial Effects of Dietary Polyphenols on Gut Microbiota and Strategies to Improve Delivery Efficiency. Nutrients.

[51] Jacoby R.P., Koprivova A., Kopriva S. (2021). Pinpointing secondary metabolites that shape the composition and function of the plant microbiome. J. Exp. Bot..

[52] Sorlozano-Puerto A., Albertuz-Crespo M., Lopez-Machado I., Ariza-Romero J.J., Baños-Arjona A., Exposito-Ruiz M., Gutierrez-Fernandez J. (2018). In Vitro Antibacterial Activity of Propyl-Propane-Thiosulfinate and Propyl-Propane-Thiosulfonate Derived from Allium spp. against Gram-Negative and Gram-Positive Multidrug-Resistant Bacteria Isolated from Human Samples. BioMed Res. Int..

[53] Putnik P., Gabrić D., Roohinejad S., Barba F.J., Granato D., Mallikarjunan K., Lorenzo J.M., Kovačević D.B. (2019). An overview of organosulfur compounds from Allium spp.: From processing and preservation to evaluation of their bioavailability, antimicrobial, and anti-inflammatory properties. Food Chem..

[54] Subramanian M.S., Ms G.N., Nordin S.A., Thilakavathy K., Joseph N. (2020). Prevailing Knowledge on the Bioavailability and Biological Activities of Sulphur Compounds from Alliums: A Potential Drug Candidate. Molecules.

[55] Batiha G.E.-S., Beshbishy A.M., Wasef L.G., Elewa Y.H.A., Al-Sagan A.A., El-Hack M.E.A., Taha A.E., Abd-Elhakim Y.M., Devkota H.P. (2020). Chemical Constituents and Pharmacological Activities of Garlic (Allium sativum L.): A Review. Nutrients.

[56] Corzomartinez M., Corzo N., Villamiel M. (2007). Biological properties of onions and garlic. Trends Food Sci. Technol..

[57] Rouf R., Uddin S.J., Sarker D.K., Islam M.T., Ali E.S., Shilpi J.A., Nahar L., Tiralongo E., Sarker S.D. (2020). Antiviral potential of garlic (Allium sativum) and its organosulfur compounds: A systematic update of pre-clinical and clinical data. Trends Food Sci. Technol..

[58] Nyhan L., Przyjalgowski M., Lewis L., Begley M., Callanan M. (2021). Investigating the Use of Ultraviolet Light Emitting Diodes (UV-LEDs) for the Inactivation of Bacteria in Powdered Food Ingredients. Foods.

[59] Lanzotti V. (2006). The analysis of onion and garlic. J. Chromatogr. A.

[60] Keusgen M., Schulz H., Glodek J., Krest I., Krüger H., Herchert N., Keller J. (2002). Characterization of SomeAlliumHybrids by Aroma Precursors, Aroma Profiles, and Alliinase Activity. J. Agric. Food Chem..

[61] Page M.J., McKenzie J.E., Bossuyt P.M., Boutron I., Hoffmann T.C., Mulrow C.D., Shamseer L., Tetzlaff J.M., Akl E.A., Brennan S.E. (2021). The PRISMA 2020 statement: An updated guideline for reporting systematic reviews. BMJ.

[62] Chen K., Nakasone Y., Xie K., Sakao K., Hou D.-X. (2020). Modulation of Allicin-Free Garlic on Gut Microbiome. Molecules.

[63] Roshan N., Riley T., Hammer K.A. (2017). Antimicrobial activity of natural products against Clostridium difficile in vitro. J. Appl. Microbiol..

[64] Roshan N., Riley T.V., Knight D.R., Hammer K.A. (2018). Effect of natural products on the production and activity of Clostridium difficile toxins in vitro. Sci. Rep..

[65] Ruiz R., García M., Lara A., Rubio L. (2010). Garlic derivatives (PTS and PTS-O) differently affect the ecology of swine faecal microbiota in vitro. Vet. Microbiol..

[66] Zhai B., Zhang C., Sheng Y., Zhao C., He X., Xu W., Huang K., Luo Y. (2018). Hypoglycemic and hypolipidemic effect of S-allyl-cysteine sulfoxide (alliin) in DIO mice. Sci. Rep..

[67] Vezza T., Algieri F., Garrido-Mesa J., Utrilla M.P., Rodríguez-Cabezas M.E., Baños A., Guillamón E., García F., Rodríguez-Nogales A., Galvez J. (2019). The Immunomodulatory Properties of Propyl-Propane Thiosulfonate Contribute to its Intestinal Anti-Inflammatory Effect in Experimental Colitis. Mol. Nutr. Food Res..

[68] Zhang C., Xie J., Li X., Luo J., Huang X., Liu L., Peng X. (2019). Alliin alters gut microbiota and gene expression of colonic epithelial tissues. J. Food Biochem..

[69] Chen K., Xie K., Liu Z., Nakasone Y., Sakao K., Hossain A., Hou D.-X. (2019). Preventive Effects and Mechanisms of Garlic on Dyslipidemia and Gut Microbiome Dysbiosis. Nutrients.

[70] Peinado M.J., Ruiz R., Echávarri A., Rubio L.A. (2012). Garlic derivative propyl propane thiosulfonate is effective against broiler enteropathogens in vivo. Poult. Sci..

[71] Peinado M., Ruiz R., Echávarri A., Aranda-Olmedo I., Rubio L. (2013). Garlic derivative PTS-O modulates intestinal microbiota composition and improves digestibility in growing broiler chickens. Anim. Feed. Sci. Technol..

[72] Rubio L.A., Peinado M.J., Ruiz R., Suárez-Pereira E., Mellet M.D.C.O., Fernández J.M.G. (2014). Correlations between changes in intestinal microbiota composition and performance parameters in broiler chickens. J. Anim. Physiol. Anim. Nutr..

[73] Ruiz R., Peinado M.J., Aranda-Olmedo I., Abecia L., Suárez-Pereira E., Mellet C.O., Fernández J.M.G., Rubio L.A. (2015). Effects of feed additives on ileal mucosa–associated microbiota composition of broiler chickens1. J. Anim. Sci..

[74] Abad P., Arroyo-Manzanares N., Ariza J.J., Baños A., García-Campaña A.M. (2020). Effect of Allium Extract Supplementation on Egg Quality, Productivity, and Intestinal Microbiota of Laying Hens. Animals.

[75] Sánchez C.J., Martínez-Miró S., Ariza J.J., Madrid J., Orengo J., Aguinaga M.A., Baños A., Hernández F. (2020). Effect of *Alliaceae* Extract Supplementation on Performance and Intestinal Microbiota of Growing-Finishing Pig. Animals.

[76] Rabelo-Ruiz M., Ariza-Romero J., Zurita-González M., Martín-Platero A., Baños A., Maqueda M., Valdivia E., Martínez-Bueno M., Peralta-Sánchez J. (2021). *Allium*-Based Phytobiotic Enhances Egg Production in Laying Hens through Microbial Composition Changes in Ileum and Cecum. Animals.

[77] Rabelo-Ruiz M., Teso-Pérez C., Peralta-Sánchez J., Ariza J., Martín-Platero A., Casabuena-Rincón Ó., Vázquez-Chas P., Guillamón E., Aguinaga-Casañas M., Maqueda M. (2021). *Allium* Extract Implements Weaned Piglet’s Productive Parameters by Modulating Distal Gut Microbiota. Antibiotics.

[78] Satora M., Magdziarz M., Rząsa A., Rypuła K., Płoneczka-Janeczko K. (2020). Insight into the intestinal microbiome of farrowing sows following the administration of garlic (Allium sativum) extract and probiotic bacteria cultures under farming conditions. BMC Vet. Res..

[79] González I.F., Urrútia G., Alonso-Coello P. (2011). Revisiones sistemáticas y metaanálisis: Bases conceptuales e interpretación. Rev. Española Cardiol..

[80] Ortiz-Martínez V.M., Andreo-Martínez P., García-Martínez N., Rios A.P.D.L., Hernández-Fernández F.J., Quesada-Medina J. (2019). Approach to biodiesel production from microalgae under supercritical conditions by the PRISMA method. Fuel Process. Technol..

[81] Zhao L., Zhang Q., Ma W., Tian F., Shen H., Zhou M. (2017). A combination of quercetin and resveratrol reduces obesity in high-fat diet-fed rats by modulation of gut microbiota. Food Funct..

[82] Shao X., Sun C., Tang X., Zhang X., Han D., Liang S., Qu R., Hui X., Shan Y., Hu L. (2020). Anti-Inflammatory and Intestinal Microbiota Modulation Properties of Jinxiang Garlic (*Allium sativum* L.) Polysaccharides toward Dextran Sodium Sulfate-Induced Colitis. J. Agric. Food Chem..

[83] Si X.-B., Zhang X.-M., Wang S., Lan Y., Zhang S., Huo L.-Y. (2019). Allicin as add-on therapy for Helicobacter pylori infection: A systematic review and meta-analysis. World J. Gastroenterol..

[84] Van Immerseel F., De Buck J., Pasmans F., Huyghebaert G., Haesebrouck F., Ducatelle R. (2004). Clostridium perfringensin poultry: An emerging threat for animal and public health. Avian Pathol..

[85] Rajput D.S., Zeng D., Khalique A., Rajput S.S., Wang H., Zhao Y., Sun N., Ni X. (2020). Pretreatment with probiotics ameliorate gut health and necrotic enteritis in broiler chickens, a substitute to antibiotics. AMB Express.

[86] Gholamiandehkordi A., Eeckhaut V., Lanckriet A., Timbermont L., Bjerrum L., Ducatelle R., Haesebrouck F., Van Immerseel F. (2009). Antimicrobial resistance in Clostridium perfringens isolates from broilers in Belgium. Vet. Res. Commun..

[87] Sorlozano-Puerto A., Albertuz-Crespo M., Lopez-Machado I., Gil-Martinez L., Ariza-Romero J.J., Maroto-Tello A., Baños-Arjona A., Gutierrez-Fernandez J. (2020). Antibacterial and Antifungal Activity of Propyl-Propane-Thiosulfinate and Propyl-Propane-Thiosulfonate, Two Organosulfur Compounds from *Allium cepa*: In Vitro Antimicrobial Effect via the Gas Phase. Pharmaceuticals.

[88] Barko P., McMichael M., Swanson K., Williams D. (2017). The Gastrointestinal Microbiome: A Review. J. Vet. Intern. Med..

[89] Honda K., Littman D.R. (2016). The microbiota in adaptive immune homeostasis and disease. Nat. Cell Biol..

[90] Weiss G.A., Hennet T. (2017). Mechanisms and consequences of intestinal dysbiosis. Cell. Mol. Life Sci..

[91] Levy M., Kolodziejczyk A., Thaiss C.A., Elinav E. (2017). Dysbiosis and the immune system. Nat. Rev. Immunol..

[92] Liu L., Zhao X., Liu Y., Zhao H., Li F. (2019). Dietary addition of garlic straw improved the intestinal barrier in rabbits1. J. Anim. Sci..

[93] Cardinal K.M., Kipper M., Andretta I., Ribeiro A.M.L. (2019). Withdrawal of antibiotic growth promoters from broiler diets: Performance indexes and economic impact. Poult. Sci..

[94] Lillehoj H., Liu Y., Calsamiglia S., Fernandez-Miyakawa M.E., Chi F., Cravens R.L., Oh S., Gay C.G. (2018). Phytochemicals as antibiotic alternatives to promote growth and enhance host health. Vet. Res..

[95] Kothari D., Lee W.-D., Niu K.-M., Kim S.-K. (2019). The Genus Allium as Poultry Feed Additive: A Review. Animals.

[96] Rahman S.U., Khan S., Chand N., Sadique U., Khan R.U. (2017). In vivo effects of Allium cepa L. on the selected gut microflora and intestinal histomorphology in broiler. Acta Histochem..

[97] Gong H., Wu M., Lang W., Yang M., Wang J., Wang Y., Zhang Y., Zheng X. (2020). Effects of laying breeder hens dietary β-carotene, curcumin, allicin, and sodium butyrate supplementation on the growth performance, immunity, and jejunum morphology of their offspring chicks. Poult. Sci..

[98] Pourabedin M., Zhao X. (2015). Prebiotics and gut microbiota in chickens. FEMS Microbiol. Lett..

[99] Wang H., Xu R., Zhang H., Su Y., Zhu W. (2020). Swine gut microbiota and its interaction with host nutrient metabolism. Anim. Nutr..

[100] De Rodas B., Youmans B.P., Danzeisen J.L., Tran H., Johnson T.J. (2018). Microbiome profiling of commercial pigs from farrow to finish. J. Anim. Sci..

[101] Lee S.-H., Bang S., Jang H.-H., Lee E.-B., Kim B.-S., Kim S.-H., Kang S.-H., Lee K.-W., Kim D.-W., Kim J.-B. (2020). Effects of Allium hookeri on gut microbiome related to growth performance in young broiler chickens. PLoS ONE.

